# Quality of Trauma Meeting Presentations Before and After Introduction of a Structured Template at a Major Trauma Centre: A Before-and-After Quality Improvement Study

**DOI:** 10.7759/cureus.111299

**Published:** 2026-06-22

**Authors:** Hamza Ahmed, Marium Rizwan, Abed Alfattah Mahmoud Alnsour, Abdur Rehman, Mohamed Said Ammar, Maham Ijaz

**Affiliations:** 1 Trauma and Orthopaedics, Salford Royal NHS Foundation Trust, Salford, GBR; 2 Spinal Surgery, Salford Royal NHS Foundation Trust, Salford, GBR

**Keywords:** multidisciplinary team, orthopaedic handover, patient safety, structured template, trauma meeting

## Abstract

Background: Morning trauma meetings are central to the daily trauma and orthopaedic workflow in major trauma centres. When case presentations are inconsistent, important clinical details may be missed, clarification questions increase, decision-making becomes less efficient, and documentation of management plans can vary. Structured presentation tools, including acronym-based templates embedded in digital systems, may improve reliability while still allowing clinicians to use judgement.

Objective: The objective of this study is to evaluate whether introducing a structured trauma meeting presentation template improved the quality, completeness, and efficiency of case presentations at a major trauma centre.

Methods: We conducted a single-centre before-and-after quality improvement study of consecutive trauma meeting case presentations between September 2025 and January 2026. A 10-domain, 20-point presentation quality score was developed using local governance priorities and published handover principles. Baseline presentations were observed before the intervention. A one-page structured template was then introduced, supported by brief induction teaching and display of the template in the trauma meeting room. The primary outcome was the mean presentation quality score. Secondary outcomes included domain-level completeness, number of clarification prompts, proportion of presentations with a documented management plan, and median presentation duration. Between-group comparisons were performed using the independent samples t-test, Mann-Whitney U test, chi-square test, or Fisher’s exact test, as appropriate.

Results: A total of 150 presentations were assessed, with 75 before and 75 after template implementation. The mean quality score increased from 11.6 ± 3.0 to 17.1 ± 2.1 out of 20, giving a mean difference of 5.5 points (95% CI: 4.7-6.3; p<0.001). Complete presentations, defined a priori as a score of 16 or above, increased from 18/75 (24.0%) to 61/75 (81.3%; p<0.001). Presentations with a documented management plan increased from 49/75 (65.3%) to 72/75 (96.0%; p<0.001), while task allocation increased from 28/75 (37.3%) to 67/75 (89.3%; p<0.001). Median clarification prompts per presentation decreased from 2 (IQR: 1-3) to 1 (IQR: 0-1; p<0.001). Median presentation duration was not significantly changed, decreasing slightly from 2.7 to 2.5 minutes (p=0.18).

Conclusion: Introducing a structured trauma meeting presentation template, expanded from an acronym within the electronic system, was associated with improved information completeness, clearer management planning, and fewer clarification prompts without prolonging meetings. These findings support structured, locally adapted templates as a low-cost intervention to improve the reliability of trauma meeting presentations in high-volume major trauma centres.

## Introduction

The morning trauma meeting is a key operational and clinical governance process in trauma and orthopaedic surgery [1]. In a major trauma centre, this meeting often shapes theatre prioritisation, consultant-led treatment decisions, further imaging, subspecialty referral, discharge planning, and escalation of deteriorating patients. Its value depends on the clear presentation of relevant information to the right multidisciplinary audience [2]. When key details are missed, decisions may be delayed, clinicians may need to search through electronic records or imaging systems during the meeting, and the risk of miscommunication increases.

Clinical handover is widely recognised as a vulnerable point in the patient journey [3]. Surgical handover is particularly challenging because decisions are often time-critical, plans may involve several teams, and patient volume can be high. Trauma and orthopaedic studies have suggested that unstructured handover may contribute to inefficiency, delayed operations, delayed discharge, and potential patient harm [4]. Human-factors work in orthopaedic handover has also highlighted the impact of environmental distractions, variable attendance, inconsistent structure, and absence of key personnel on the quality of information transfer.

Structured templates and checklists are useful in this setting because they make expectations explicit. They do not replace clinical judgement, but they provide a shared framework for concise and consistent communication. They also help the meeting chair compare cases using a common set of clinical details. For a trauma meeting, a template needs to be brief enough to avoid slowing the discussion while still prompting high-value information. This includes patient identifiers, diagnosis, imaging, neurovascular status, comorbidities, anticoagulation, open injury or soft-tissue concerns, theatre priority, senior decision-maker, management plan, and allocation of responsibility [5].

The need for reliable communication is even greater in major trauma centres, where daily patient flow may include emergency department referrals, inter-hospital transfers, high-energy injuries, polytrauma, complex peri-articular fractures, open fractures, frailty-related injuries, and cases requiring input from orthoplastics, anaesthetics, geriatrics, radiology, intensive care, rehabilitation, and specialist nursing [6]. Presentation quality therefore has implications beyond the meeting itself. Poor information transfer can affect operative sequencing, pre-operative optimisation, documentation quality, and the clarity of tasks handed over to junior doctors, trauma coordinators, and other members of the team [7].

Previous studies have reported improvements in trauma handover after the introduction of proformas or structured protocols. However, many hospitals continue to rely on variable local practice. In our major trauma centre, informal feedback from consultants, trainees, trauma coordinators, and theatre staff suggested that morning trauma meeting presentations varied in structure and often led to repeated clarification questions. This quality improvement project therefore evaluated whether introducing a structured trauma meeting presentation template improved the completeness, quality, and efficiency of case presentations.

The primary aim was to determine whether the template improved the overall presentation quality score. Secondary aims were to assess changes in domain-level information completeness, frequency of clarification prompts, documentation of a management plan, allocation of tasks and responsibility, and presentation duration. Presentation duration was also used as a balancing measure to assess whether the template disrupted the flow of the meeting or made individual case presentations longer.

## Materials and methods

Study design and setting

This was a single-centre before-and-after quality improvement study conducted in the Department of Trauma and Orthopaedic Surgery at a major trauma centre in the United Kingdom. The project was designed using the plan-do-study-act approach and reported in a format consistent with quality improvement and observational study reporting principles. The morning trauma meeting was held each weekday and attended by the on-call consultant trauma surgeon, orthopaedic registrars, junior doctors, trauma coordinators, theatre scheduling staff, and representatives from relevant subspecialty teams depending on the caseload.

Study period and population

Consecutive case presentations at the morning trauma meeting were assessed over two phases between September 2025 and January 2026. The baseline phase included 75 presentations observed before template introduction from 1st September 2025 to 16th November 2025. The post-intervention phase included 75 presentations after implementation from 18th January 2026 to 31st January 2026. Presentations were eligible if they related to an acute trauma patient discussed for operative decision-making, non-operative management planning, inter-specialty referral, imaging review, repatriation, or discharge planning. Elective cases, purely administrative updates without a patient-specific clinical decision, duplicated presentations of the same patient on the same day, and cases discussed outside the formal trauma meeting were excluded.

Intervention

The intervention was a structured trauma meeting presentation template that expanded from an acronym on the electronic patient record system and was developed by a registrar after reviewing local governance priorities and published handover principles. The template was deliberately concise and contained 10 domains: identifiers, mechanism/injury, imaging, neurovascular status, comorbidities/anticoagulation, open injury or soft-tissue status, urgency/theatre priority, named senior decision-maker, management plan, and tasks/responsibility. Table 1 presents the structured trauma meeting presentation template domains and scoring rubric used in this study. It was introduced through a five-minute briefing at the end of a departmental governance meeting, laminated copies were placed in the trauma meeting room, and a version was added to the junior doctor induction pack and handover folder.

**Table 1:** Structured trauma meeting presentation template domains and scoring rubric The structured trauma meeting presentation template was developed by the authors of the present study using local governance priorities and published handover principles.

No.	Domain	Minimum expected information	Score
1	Patient identifiers	Name, age/date of birth, hospital number, ward/location, side/site	0 absent; 1 partial; 2 complete
2	Mechanism/injury summary	Mechanism, diagnosis, fracture classification where relevant	0 absent; 1 partial; 2 complete
3	Relevant imaging reviewed	Plain radiographs/CT/MRI reviewed and key finding stated	0 absent; 1 partial; 2 complete
4	Neurovascular status	Distal neurovascular status documented for limb injuries	0 absent; 1 partial; 2 complete
5	Comorbidities/anticoagulation	Pertinent comorbidity, frailty, anticoagulant/antiplatelet status	0 absent; 1 partial; 2 complete
6	Open injury/soft-tissue status	Open/closed injury, wounds, skin compromise, compartment concern	0 absent; 1 partial; 2 complete
7	Theatre priority/urgency	Need for theatre, urgency category, fasting status if known	0 absent; 1 partial; 2 complete
8	Named senior decision-maker	Consultant or senior registrar responsible for plan	0 absent; 1 partial; 2 complete
9	Documented management plan	Operative/non-operative plan, further imaging, review, discharge pathway	0 absent; 1 partial; 2 complete
10	Tasks and responsibilities allocated	Named person/team for consent, imaging, pre-op workup, referrals	0 absent; 1 partial; 2 complete

Outcome measures

The primary outcome was a presentation quality score from 0 to 20. Each of the 10 domains was scored as 0 if absent, 1 if partially communicated, and 2 if clearly communicated. A complete presentation was defined a priori as a score of 16 or above. Secondary outcomes were the presence of each domain, the number of clarification prompts from the chair or multidisciplinary team, the presence of a documented management plan, task allocation to a named person or role, and presentation duration measured from the start of the case presentation to the chair moving to the next case. Clarification prompts were defined as questions seeking essential information that would have been covered by the template.

Data collection

Two observers (Ahmed, Rizwan), both familiar with the trauma meeting process, piloted the scoring tool on 10 presentations not included in the final analysis. During data collection, one observer scored each presentation in real time using a standardised data collection sheet. A random 20% sample was independently scored by a second observer to assess inter-rater agreement. Disagreements were resolved by discussion after the meeting using contemporaneous notes and electronic records where necessary. No patient-identifiable information was stored in the spreadsheet.

Statistical analysis

Continuous variables were tested for approximate normality by visual inspection of histograms and summary statistics. Mean quality scores were compared using an independent samples t-test and presented with 95% confidence intervals. Non-normally distributed variables, including clarification prompts and presentation duration, were compared using the Mann-Whitney U test. Categorical variables were compared using chi-square or Fisher's exact testing. A two-sided p-value of <0.05 was considered statistically significant. Analyses were performed using standard spreadsheet functions and cross-checked in IBM SPSS Statistics for Windows, Version 32 (Released 2026; IBM Corp., Armonk, New York, United States). Inter-rater agreement for the binary complete/incomplete classification was summarised using Cohen's kappa.

## Results

A total of 150 eligible trauma meeting presentations were included: 75 in the baseline phase and 75 after template implementation. No eligible presentation was excluded because of incomplete scoring. The case mix was broadly similar between phases, with lower-limb trauma, upper-limb trauma, fragility fractures, paediatric trauma, and complex/polytrauma cases represented in both groups. The post-intervention phase contained slightly more lower-limb and complex cases, but the difference was not statistically significant. Table 2 summarises the case presentation characteristics before and after template implementation.

**Table 2:** Case presentation characteristics before and after template implementation

Characteristic	Pre-template	Post-template	Overall
Total presentations assessed	75	75	150
Adult presentations	68 (90.7%)	67 (89.3%)	135 (90.0%)
Paediatric presentations	7 (9.3%)	8 (10.7%)	15 (10.0%)
Lower-limb trauma	31 (41.3%)	34 (45.3%)	65 (43.3%)
Upper-limb trauma	24 (32.0%)	22 (29.3%)	46 (30.7%)
Fragility fracture pathway case	12 (16.0%)	11 (14.7%)	23 (15.3%)
Complex/polytrauma or open injury	8 (10.7%)	8 (10.7%)	16 (10.7%)
Presented by foundation/core trainee	49 (65.3%)	51 (68.0%)	100 (66.7%)
Presented by registrar or advanced practitioner	26 (34.7%)	24 (32.0%)	50 (33.3%)

Inter-rater agreement was assessed in 30 presentations. Agreement for the complete versus incomplete presentation classification was high, with a Cohen's kappa of 0.82. Agreement for individual domains ranged from 0.74 for comorbidities/anticoagulation to 0.91 for documented management plan.

Primary outcome 

The mean presentation quality score improved from 11.6 ± 3.0 before introduction of the template to 17.1 ± 2.1 after implementation. This represented a mean increase of 5.5 points, with a 95% confidence interval of 4.7 to 6.3 points, and was statistically significant (p<0.001). In relative terms, the mean score increased by 47.4%. The proportion of presentations meeting the predefined threshold for completeness increased from 18/75 (24.0%) to 61/75 (81.3%; p<0.001). Table 3 presents the primary and secondary outcomes before and after template implementation.

**Table 3:** Primary and secondary outcomes Pre-template presentations, n=75; post-template presentations, n=75. Continuous normally distributed data were compared using the independent samples t-test. Non-normally distributed data were compared using the Mann-Whitney U test. Categorical data were compared using the chi-square test or Fisher’s exact test, as appropriate.

Outcome	Pre-template, n=75	Post-template, n=75	Absolute change	Statistical test	Test statistic	p-value
Mean quality score, mean ± SD	11.6 ± 3.0	17.1 ± 2.1	+5.5 points	Independent samples t-test	t=13.01	<0.001
Complete presentation (score >=16), n (%)	18 (24.0%)	61 (81.3%)	+57.3 percentage points	Chi-square test	χ²=49.45	<0.001
Documented management plan, n (%)	49 (65.3%)	72 (96.0%)	+30.7 percentage points	Chi-square test	χ²=22.61	<0.001
Tasks/responsibility allocated, n (%)	28 (37.3%)	67 (89.3%)	+52.0 percentage points	Chi-square test	χ²=43.67	<0.001
Clarification prompts per presentation, median (IQR)	2 (1-3)	1 (0-1)	-1 prompt	Mann-Whitney U test	U=913	<0.001
Presentation duration, minutes, median (IQR)	2.7 (2.2-3.4)	2.5 (2.1-3.1)	-0.2 minutes	Mann-Whitney U test	U=2456	0.18
Cases needing electronic record opened for missing details, n (%)	34 (45.3%)	9 (12.0%)	-33.3 percentage points	Chi-square test	χ²=20.38	<0.001
Cases deferred for missing information, n (%)	8 (10.7%)	1 (1.3%)	-9.4 percentage points	Fisher’s exact test	OR=8.84	0.034

Secondary outcomes

Improvements were seen across all 10 presentation domains. The largest absolute gains were observed in task allocation, which increased by 52.0 percentage points; open injury or soft-tissue status, which increased by 42.7 percentage points; named senior decision-maker, which increased by 42.7 percentage points; and neurovascular status, which also increased by 42.7 percentage points. Clarification prompts decreased after implementation, and management plans were documented more consistently. Importantly, presentation duration did not increase.

**Figure 1:**
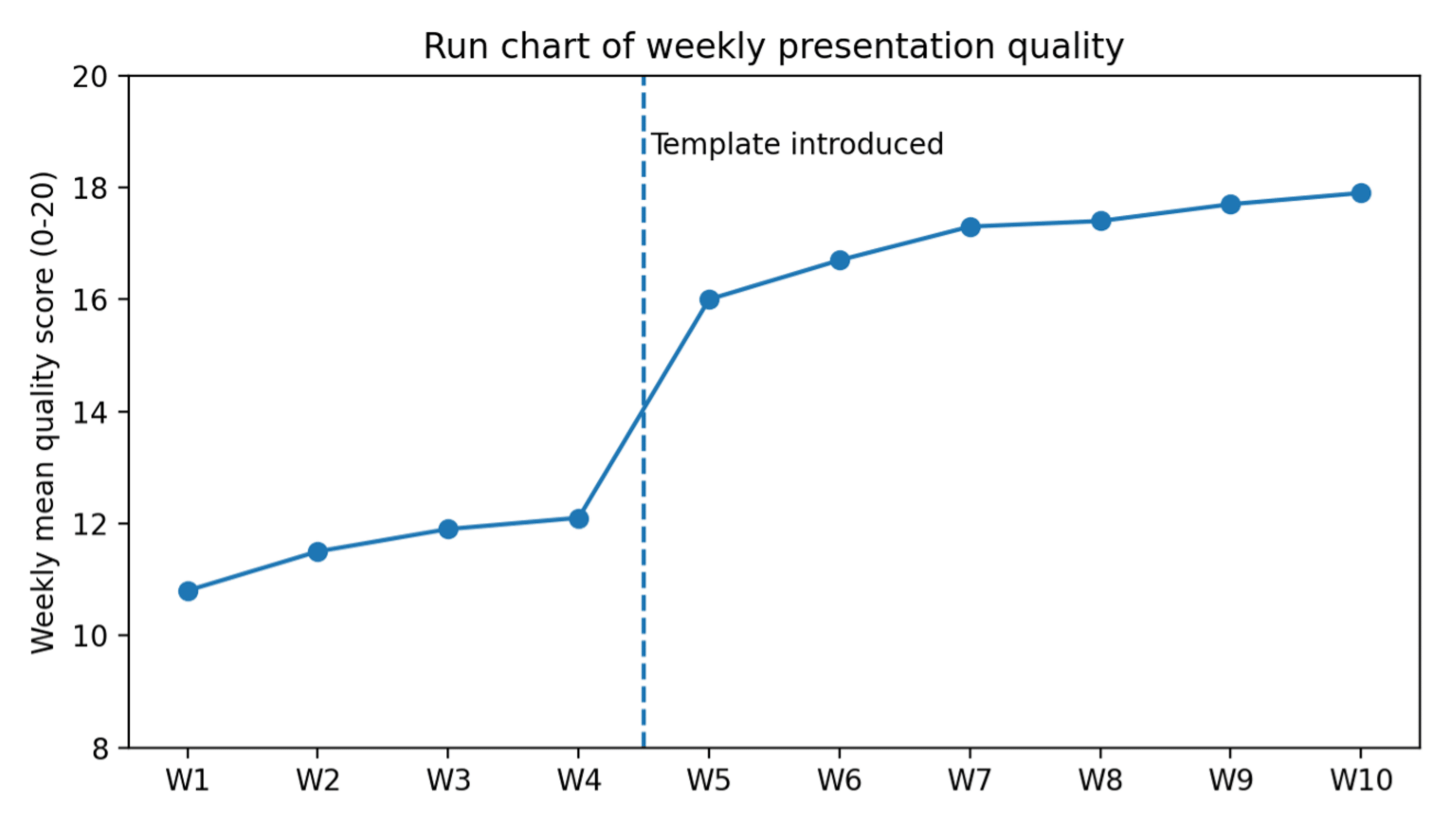
Weekly mean presentation quality score. The dashed line indicates introduction of the structured template after week 4 Each weekly value represents the mean presentation quality score for the number of presentations evaluated per week (seven). Plotted values represent mean scores.

Figure 1 is a run chart showing weekly mean presentation quality scores over time. The scores were relatively low and only gradually improving during the baseline period, rising from about 10.8 in week 1 to 12.1 in week 4. The dashed vertical line marks the point at which the structured trauma meeting template was introduced. After implementation, there was a clear step-up in performance, with the weekly mean score increasing to about 16.0 in week 5 and then continuing to improve gradually to 17.9 by week 10.

Completeness improved across all 10 presentation domains after introduction of the structured template. Documentation of patient identifiers was already high before implementation and increased from 66/75 (88.0%) to 75/75 (100.0%). Other core presentation elements also improved, including mechanism or injury summary, which increased from 59/75 (78.7%) to 73/75 (97.3%), and relevant imaging reviewed, which increased from 62/75 (82.7%) to 74/75 (98.7%).

The largest gains were seen in domains that were less consistently communicated at baseline. Task and responsibility allocation increased from 28/75 (37.3%) to 67/75 (89.3%), representing the greatest absolute improvement at 52.0 percentage points. Neurovascular status improved from 34/75 (45.3%) to 66/75 (88.0%), and open injury or soft-tissue status improved from 31/75 (41.3%) to 63/75 (84.0%). Documentation of a named senior decision-maker increased from 37/75 (49.3%) to 68/75 (90.7%), while comorbidities and anticoagulation increased from 42/75 (56.0%) to 69/75 (92.0%). Theatre priority or urgency increased from 46/75 (61.3%) to 71/75 (94.7%), and documented management plans increased from 49/75 (65.3%) to 72/75 (96.0%).

Overall, the greatest improvements occurred in action-focused and safety-critical domains, particularly task allocation, neurovascular status, open injury or soft-tissue status, and identification of the senior decision-maker. Table 4 shows the domain-level documentation completeness before and after template implementation.

**Table 4:** Domain-level documentation completeness Pre-template presentations, n=75; post-template presentations, n=75. Values are presented as N (%).

Domain	Pre-template, n=75	Post-template, n=75	Absolute change
Patient identifiers	66 (88.0%)	75 (100.0%)	+12.0 percentage points
Mechanism/injury summary	59 (78.7%)	73 (97.3%)	+18.6 percentage points
Relevant imaging reviewed	62 (82.7%)	74 (98.7%)	+16.0 percentage points
Neurovascular status	34 (45.3%)	66 (88.0%)	+42.7 percentage points
Comorbidities/anticoagulation	42 (56.0%)	69 (92.0%)	+36.0 percentage points
Open injury/soft-tissue status	31 (41.3%)	63 (84.0%)	+42.7 percentage points
Theatre priority/urgency	46 (61.3%)	71 (94.7%)	+33.4 percentage points
Named senior decision-maker	37 (49.3%)	68 (90.7%)	+41.4 percentage points
Documented management plan	49 (65.3%)	72 (96.0%)	+30.7 percentage points
Tasks and responsibility allocated	28 (37.3%)	67 (89.3%)	+52.0 percentage points

## Discussion

This before-and-after quality improvement study found that a simple structured trauma meeting presentation template was associated with a clear improvement in the quality and completeness of case presentations at a major trauma centre. The mean quality score increased by 5.5 points on a 20-point scale, and the proportion of complete presentations increased more than threefold. Importantly, this improvement was achieved without increasing median presentation duration, suggesting that the template improved the density and reliability of information transfer rather than adding extra administrative burden.

The largest improvements were seen in areas that are easily missed during a busy trauma meeting: task allocation, open injury or soft-tissue status, named senior decision-maker, and neurovascular status. These details have direct clinical relevance. Failure to state the neurovascular status may obscure urgency [8]. Failure to describe an open injury or soft-tissue compromise may delay antibiotics, plastics input, or theatre prioritisation [9]. Similarly, failure to allocate tasks clearly can leave uncertainty after the meeting has ended [10]. Improved task allocation is particularly important in major trauma centres, where responsibility may be shared between on-call teams, subspecialty services, theatre coordinators, radiology, and inpatient wards [11].

Our findings are consistent with the wider patient-safety literature showing that structured handover tools can improve the reliability of information transfer. Orthopaedic-specific work has also highlighted the value of structure in trauma handover, with protocols and proformas improving the completeness of information exchanged during morning trauma meetings [12]. This project adds a focused assessment of verbal presentation quality during the meeting itself, rather than assessing only written documentation or out-of-hours handover. It also shows that a concise template can be incorporated into routine high-volume practice without slowing the meeting.

The improvement in documented management plans and the reduction in clarification prompts are especially relevant from an operational perspective [13]. Clarification questions are sometimes necessary and clinically valuable, but repeated questions about basic missing information can slow the meeting and increase cognitive load. A well-designed template allows the chair and multidisciplinary team to spend less time retrieving missing facts and more time making decisions. In our sample, fewer cases required the electronic record to be opened to find missing information, and fewer decisions were deferred because essential details were absent.

Several practical features probably supported implementation. The template was short, visible, and designed around the needs of the clinicians using it. It did not require a new electronic system, extra staffing, or lengthy training. It was introduced as a communication aid rather than as an additional form to complete. This distinction matters because checklist fatigue can limit uptake when tools are too long, repetitive, or poorly integrated into daily workflow [14]. In contrast, the 10-domain template represented a minimum useful dataset for local trauma decision-making and could be delivered verbally in under three minutes.

This project also has implications for clinical governance and trainee education. Morning trauma meetings are often attended by rotating junior doctors who may be unfamiliar with consultant expectations or local pathways. A structured template provides a shared standard that can be taught during induction and reinforced through feedback. It may also support future work on improving written documentation, theatre prioritisation, referral quality, and patient flow. Because the intervention is low-cost and easy to adapt, it could be modified for use in district general hospitals, trauma units, and other major trauma centres, including settings with dedicated paediatric, spinal, pelvic, orthoplastic, or frailty-trauma pathways.

This study has several strengths. It evaluated consecutive real-time trauma meeting presentations, which reduced selection bias within the observed periods. The scoring rubric was defined before data collection and covered clinically relevant domains identified from local governance priorities and published handover principles. A balancing measure was included to ensure that improved presentation quality was not achieved simply by making presentations longer. The intervention itself was simple, low-cost, and practical for implementation in a busy major trauma centre.

There are also limitations. The before-and-after design is vulnerable to secular trends, Hawthorne effect, and changes in case mix or staffing between audit cycles. The presentation quality score was locally developed and has not been externally validated, although it was based on clinically relevant domains and showed high inter-rater agreement in this sample. The study measured process outcomes rather than patient-level outcomes such as time to theatre, complications, length of stay, or readmission. It was also conducted at a single major trauma centre, and the exact template may need adaptation before use in other institutions. Finally, sustained compliance beyond the initial post-intervention period was not assessed, so future audit cycles should evaluate whether improvement is maintained after trainee rotation and induction changes.

Future work should include re-audit after six to 12 months, assessment of weekend or out-of-hours meetings, linkage with written documentation quality, and exploration of patient-centred outcomes. Further study could also examine whether structured presentation training improves junior doctors' confidence and reduces variation between presenters.

## Conclusions

At a major trauma centre, introducing a structured trauma meeting presentation template was associated with a substantial improvement in the quality and completeness of case presentations. Management plans and allocated tasks were documented more consistently, and fewer clarification prompts were required during the meeting. Importantly, these improvements were achieved without prolonging presentations, and the template was feasible to implement using existing departmental resources.

Structured trauma meeting templates should be considered as a practical patient-safety and workflow intervention, particularly in high-volume trauma and orthopaedic departments where rotating junior staff present cases to a multidisciplinary team.

## Appendices

Appendix A. Structured trauma meeting presentation template used for the intervention

1. Patient identifiers: name, age/date of birth, hospital number, location, side/site.

2. Mechanism and injury: concise mechanism, diagnosis, relevant classification.

3. Imaging: radiographs/CT/MRI obtained and key findings.

4. Neurovascular status: intact/abnormal/not assessable and reason.

5. Medical context: frailty, comorbidities, anticoagulation/antiplatelets, anaesthetic concerns.

6. Soft tissue/open injury: open/closed, wounds, skin compromise, antibiotics/tetanus where relevant.

7. Urgency: theatre priority, fasting status, need for specialist input.

8. Senior decision-maker: consultant or senior registrar responsible.

9. Plan: operative/non-operative, imaging, admission/discharge, transfer/repatriation.

10. Tasks: named person/team responsible for consent, booking, referrals, documentation, family update.
